# Therapeutic effect of aged garlic extract on gingivitis in dogs

**DOI:** 10.3389/fvets.2023.1277272

**Published:** 2023-11-06

**Authors:** Kaori Takahashi, Hiroshi Nango, Mitsuyasu Ushijima, Miyuki Takashima, Masato Nakamoto, Toshiaki Matsutomo, Hiroshi Jikihara, Nanami Arakawa, Shinichiro Maki, Akira Yabuki, Yasuyuki Endo, Osamu Yamato

**Affiliations:** ^1^Kagoshima University Veterinary Teaching Hospital, Joint Faculty of Veterinary Medicine, Kagoshima University, Kagoshima, Japan; ^2^Central Research Institute, Wakunaga Pharmaceutical Co., Ltd., Hiroshima, Japan; ^3^Research Administration Department, Wakunaga Pharmaceutical Co., Ltd., Hiroshima, Japan; ^4^Laboratory of Clinical Pathology, Department of Veterinary Medicine, Joint Faculty of Veterinary Medicine, Kagoshima University, Kagoshima, Japan

**Keywords:** aged garlic extract, dog, oral health, oral hygiene, dental homecare, gingival index, halitosis, salivary cathelicidin

## Abstract

Periodontal disease is one of the most common dental health problems in dogs. Clinical studies in humans have shown that aged garlic extract (AGE), which contains stable and water-soluble sulfur-containing bioactive compounds, improves the symptoms of periodontal diseases. Our previous study demonstrated that oral administration of AGE in healthy Beagle dogs at 90 mg/kg/day for 12 weeks had no adverse effects such as hemolytic anemia, which is well known to occur as a result of ingestion of *Allium* species, including onions and garlic, in dogs. However, the therapeutic potential of AGE in canine periodontal disease remains unclear. Accordingly, we investigated the therapeutic effects of AGE in Beagle dogs with mild gingivitis. Feeding 18 mg/kg/day of AGE for 8 weeks resulted in the improvement of gingival index score, level of volatile sulfur compounds in exhaled air, and enzyme activity of periodontal pathogens without any adverse effects on clinical signs and hematological and serum biochemical parameters. Moreover, AGE increased the concentration of salivary cathelicidin, an antimicrobial peptide that contributes to the oral innate immune response. These results suggest that AGE could be a potential therapeutic agent for canine gingivitis.

## 1. Introduction

Periodontal disease, a set of dental inflammatory diseases initiated by oral microbiota on the tooth surface (1), is one of the most common health problems in dogs (2). The prevalence of this disease is 44–100% across all dog breeds, based on clinical assessment and necropsy sample reports (3). In general, periodontal disease is more prevalent in small dog breeds than in large ones (3, 4), and its incidence is strongly correlated with age (4–6). Gingivitis is characterized by reversible inflammation and gingival redness without the loss of connective tissue attachment or alveolar bone. If this condition is left untreated, most, but not all, cases of gingivitis progress to periodontitis. Periodontitis is a severe chronic inflammation of the supporting tooth tissues that causes loss of connective tissue attachment and alveolar bone, possibly resulting in gingival recession, oronasal fistula, radicular abscess, tooth mobility, and tooth loss (7). Periodontitis is caused by bacterial invasion and bacterial toxins, but the extent of the disease is changed by host immune reaction that depends on genetic, immunological, and environmental factors (8, 9). Furthermore, periodontal disease is associated with systemic disorders in dogs, such as cognitive dysfunction (10), cardiac disease (4, 11), and renal disease (12, 13), ultimately leading to a poor quality of life.

Controlling the accumulation of oral microbiota on tooth surfaces and subsequent gingival inflammation is important for the prevention and treatment of periodontal diseases (14). Treatment strategies for periodontal disease are generally based on homecare with tooth brushing and professional dental cleaning, dental gum, and antibiotics as needed. Daily tooth brushing is the most effective method for removing dental plaque. A previous study demonstrated that twice-daily tooth brushing for 18 months prevents the accumulation of plaque, debris, calculus, and the subsequent development of gingivitis and periodontitis in 10-month-old Beagle dogs (15). Daily tooth brushing and oral administration of antibiotics are sometimes difficult to achieve because of poor compliance, lack of technique in owners, and aggressive temperament (16–18). Therefore, more convenient oral agents for the prevention and treatment of gingivitis in pet dogs are required for owners and veterinarians.

Herbs and phytochemicals are utilized in human dentistry as antimicrobial, antiseptic, antineoplastic, antioxidant, and analgesic agents as well as for the elimination of halitosis (19, 20). Such medicinal herbal plants include aloe (*Aloe vera*), green tea (*Camellia sinensis*), turmeric (*Curcuma longa*), kalonji (*Nigella sativa*), and neem (*Azadirachta indica*) as well as garlic (*Allium sativum*), which have been suggested as an alternate remedy for oral-dental problems in humans. Herbal-based treatments are mostly safer than synthetic drugs in humans, but some serious adverse effects may be happened (19). Some compounds in fresh garlic have the potential to cause chemical burns to the skin and mucosa even in humans (21). It is well-known that intake of garlic causes hemolytic anemia due to the oxidation of erythrocytes in dogs (22).

Aged garlic extract (AGE) is one of the garlic products prepared by the soaking of garlic with aqueous ethanol for more than 10 months at room temperature (23). AGE and its bioactive sulfur-containing amino acids, *S*-allylcysteine, *S*-1-propenylcysteine, and *S*-allylmercaptocysteine, possess favorable properties such as antioxidation (24, 25), anti-inflammation (26–28), immunomodulation (29–31), and anti-cancer activities (32). Several human clinical studies have demonstrated that AGE improves hypertension (33), atherosclerosis (34), and metabolic syndrome (35). More recently, daily intake of AGE for 4 and 18 months was reported to improve the modified gingival index, gingival bleeding index, and probing pocket depth in human patients with mild-to-moderate periodontal disease (36, 37). In support of these results, AGE and its bioactive sulfur-containing amino acids suppressed tumor necrosis factor-α-induced intracellular adhesion molecule-1 expression and interleukin-6 secretion in human gingival epithelial cell line Ca9-22 (38). These results suggest that AGE suppresses gingival inflammation and the progression to periodontal disease. Furthermore, our previous study reported that oral administration of AGE at 45 and 90 mg/kg/day for 8 and 12 weeks in Beagle dogs increased the gene expression of nuclear factor erythroid 2-related factor 2 (Nrf2) and Nrf2-regulated anti-oxidant enzymes NAD(P)H quinone oxidoreductase 1 and glutamate-cysteine ligase modifier subunit in whole blood without any adverse effects, including garlic-induced hemolytic anemia caused by oxidative injury of erythrocytes (39).

Although the safety and favorable bioactivity of AGE in dogs are already clarified as mentioned above, its therapeutic potential for canine periodontal disease remains unclear. The aim of this study was to evaluate the therapeutic effects of AGE supplementation on gingivitis in dogs.

## 2. Materials and methods

### 2.1. Preparation of AGE and placebo powders

AGE was prepared as previously described (39). Sliced cloves of garlic cultivated in Japan were soaked in an ethanol/water mixture for more than 10 months at room temperature. The extract was then dried using a circulation dryer (HOH-A3; Takabayashi Rika Co., Ltd., Tokyo, Japan). The AGE powder consisted of 18.0 mg of dried AGE extract, 19.0 mg of crystalline cellulose (Ceolus UF-F702; Asahi Kasei Chemicals Corporation, Tokyo, Japan), 0.9 mg of carboxymethyl cellulose calcium (E.C.G-FA; Nichirin Chemical Industries Ltd., Itami, Japan), and 5.0 mg of agar powder (Ina Food Industry Co., Ltd., Ina, Japan). The placebo powder that consists of crystalline cellulose (37.0 mg), calcium carboxymethyl cellulose (0.9 mg), and agar powder (5.0 mg) were used. The powder was stored at 4°C until further use. Produced AGE is sometimes analyzed by high-performance liquid chromatography to confirm to have several sulfur-containing compounds, *S*-methylcysteine, *S*-allylcysteine, *S*-1-propenylcysteine, and *S*-allylmercaptocysteine, which are characteristic sulfur compounds in AGE (40).

### 2.2. Experimental animals and treatments

The animal experiment was conducted with 10 Beagle dogs (4 males and 6 females, 2–9 years old, 9.8–11.8 kg body weight) housed at the Kitayama Labes Corporation, Narita Biocenter (Narita, Japan). Before the experiments, all dogs were confirmed to be clinically healthy based on physical examination, and hematological and serum biochemical analyzes. All dogs were housed at a temperature of 23 ± 5°C and relative humidity of 55 ± 25% under a 12 h light/dark cycle (light phase from 7:00 to 19:00). The 10 dogs were divided into two groups based on the average gingival index score: the placebo-treated control group referred as Placebo group (4.6 ± 1.2 years of age, 10.76 ± 0.41 kg body weight, and the average gingival index score 0.57 ± 0.10) and the AGE-treated group referred as AGE group (3.8 ± 0.8 years of age, 10.76 ± 0.35 kg body weight, the average gingival index score 0.53 ± 0.13), with each group consisting of five dogs (2 males and 3 females). Each dog was fed 250 g of dry food (DS-A; Oriental Yeast Co., Ltd., Tokyo, Japan) sprinkled with either a 42.9 mg/kg of placebo or AGE powder (18 mg/kg dried AGE extract) once daily between 11:00 and 12:00 for 8 weeks. Water was provided *ad libitum*. After feeding, we confirmed that all dogs had consumed the food completely. All animal experiments complied with the Guidelines for Animal Experiments of Kitayama Labes Corporation and were approved by the Animal Welfare Committee of Kitayama Labes Corporation (Approval Number NBC57-024).

### 2.3. Clinical observation and measurement of body weight

The dogs were inspected every morning for clinical manifestations, such as fecal characteristics, vomiting, coat condition, and behavior during the experimental period. The dogs were weighed at baseline (1 week before treatment), and at 4 and 8 weeks after treatment. A visual inspection of the oral cavity was performed to check for teeth, oral lesions, and dental and soft issue abnormalities at baseline, and at 4 and 8 weeks after treatment.

### 2.4. Hematology and serum biochemistry

Hematological and serum biochemical analyzes were performed as previously described (39). During the interdigestive period, 4  mL of blood was collected from the cephalic vein under unanesthetized conditions at baseline and 4 and 8 weeks after treatment. Approximately half of the blood sample was miscible with ethylenediaminetetraacetic acid dipotassium salt, and used for hematological analysis (FUJIFILM VET Systems, Tokyo, Japan). Serum was obtained from the remaining half of the collected blood samples, followed by biochemical analysis using a chemical analyzer (VETSCAN VS2; Zoetis, Florham Park, NJ, United States) with a rotor (Comprehensive Diagnostic Profile; Zoetis). Serum amyloid A (SAA) concentration was determined using an automated biochemical analyzer (Pentra C200; HORIBA ABX SAS, Montpellier, France) and a particular SAA reagent for animal serum or plasma (VET-SAA “Eiken” reagent; Eiken Chemical Co. Ltd., Tokyo, Japan). The C-reactive protein (CRP) concentration was determined using a laser nephelometric immunoassay analyzer (Laser CRP-2; Arrows Co., Ltd., Osaka, Japan).

### 2.5. Evaluation of gingival index

The severity of gingivitis was evaluated using the gingival index at baseline and 4 and 8 weeks after treatment without sedation or anesthesia as previously described (41, 42). The gingival index was measured on the buccal side of I3, C, P2, P3, P4, and M1 of the maxilla and C, P2, P3, P4, and M1 of the mandible. The gingival index was scored as follows: 0, no gingival inflammation; 0.5, slight gingival inflammation (slight change in color); 1, mild gingival inflammation (clear redness and edema, but no bleeding on probing); 2, moderate gingival inflammation (strong redness, edema, and bleeding on probing); and 3, severe gingival inflammation (marked redness, edema, ulceration, and a tendency to spontaneously bleed). The scores were assessed as a blinded experiment by the same experimenter at the Kitayama Labes Corporation. This experimenter was a Junior Laboratory Animal Technician certified by the Japanese Society of Laboratory Animals, had worked for 11 years, and experienced several experiments for the evaluation of gingival index in dogs with gingivitis.

### 2.6. Measurement of volatile sulfur compounds (VSCs) levels

The VSCs levels in exhaled air were measured using a halimeter RH17K (TAIYO Instruments Inc., Osaka, Japan) at baseline and 8 weeks after treatment. The dogs were subcutaneously administrated 1 mg/kg maropitant (Cerenia; Zoetis) at 17:00 on the day before measurement for the prevention of vomiting. On the day of measurement, 0.03 mg/mL medetomidine (Dorbene; Kyoritsu Seiyaku Co., Ltd., Tokyo, Japan) and 0.3 mg/kg midazolam (Dormicum; Maruishi Pharmaceutical Co., Ltd., Osaka, Japan) were administered intramuscularly to the thigh for sedation. Oral air samples were obtained by inserting a straw connected to a halimeter into the oral cavity, and VSCs levels were measured according to the manufacturer’s recommendations. After measurement, 0.3 mg/kg atipamezole (Atipame; Kyoritsu Seiyaku Co., Ltd., Tokyo, Japan) was administered intramuscularly. Levels of thiol, a VSC, were measured in the gingival margin at C, P2, P3, and P4 in the bilateral maxilla and mandible using OraStrip (DS Pharma Animal Health Co., Ltd., Osaka, Japan) (43, 44) at baseline and 1, 2, 4, and 8 weeks after treatment. The OraStrip test was performed according to manufacturer’s instructions. The scores were assessed as a blinded experiment using the six chart colors on the accompanying sheet by the same experimenter at the Kitayama Labes Corporation.

### 2.7. Enzyme activity of periodontal pathogens

The enzyme activity of periodontal pathogens was measured using a swab in the gingival margin at C, P2, P3, and P4 in the bilateral maxilla and mandible using ADplit (Kyoritsu Seiyaku Co., Ltd., Tokyo, Japan) (45, 46) at baseline and 1, 2, 4, and 8 weeks after treatment. The score from each test in the bilateral maxilla and mandible was averaged. A score of 1–5 was assigned according to manufacturer’s instructions. The cores were assessed as a blinded experiment using the five chart colors on the accompanying sheet by the same experimenter at the Kitayama Labes Corporation.

### 2.8. Enzyme-linked immunosorbent assay (ELISA)

Saliva was collected from outside the posterior molar using a Salivette (SARSTEDT, Nümbrecht, Germany) at baseline and 4 and 8 weeks after treatment. Quantification of immunoglobin A (IgA) and cathelicidin antimicrobial peptide (CAMP) in the saliva was performed using the Canine IgA ELISA Kit (Novus Biologicals, Centennial, CO, United States) and Canine Cathelicidin Antimicrobial Peptide ELISA Kit (MyBioSource, San Diego, CA, United States), respectively, according to the manufacturer’s protocols. The absorbance of the samples was measured at 450 nm using a Multiskan GO Microplate Spectrophotometer (Thermo Scientific, Vantaa, Finland).

### 2.9. Statistical analysis

Data analyzes were performed using Kyplot 6.0 (KyensLab Inc., Tokyo, Japan). Data are expressed as mean ± standard error of the mean. Statistical significance between the Placebo and AGE groups was assessed using the Mann–Whitney U test. Statistical changes were also assessed using the Wilcoxon signed-rank test and compared with baseline. Differences at *p* < 0.05 were considered statistically significant.

## 3. Results

### 3.1. Body weight and physical conditions

All dogs consumed food without repeated vomiting during the experimental period. No significant changes in body weight were observed in the Placebo and AGE groups during the experimental period (Supplementary Table S1). Furthermore, there were no changes in clinical symptoms such as soft feces, fur shedding, reddish urine color, or abnormal behaviors in either group. There were no abnormal changes including missing and fractured teeth, oral lesions, and hydration in the oral inspections during the experimental period.

### 3.2. Hematological and serum biochemical parameters

During the experimental period, there were several significant changes compared with the baseline data and several significant differences between the Placebo and AGE groups, although there were no substantial changes and differences (Supplementary Table S1). In the hematological data, the erythrocyte count decreased significantly (*p* < 0.05) in the AGE group at 4 and 8 weeks compared to baseline. The leukocyte count decreased significantly (*p* < 0.05) in the Placebo group at 8 weeks and in the AGE group at 4 weeks compared to baseline. The monocyte count decreased significantly (*p* < 0.05) in both groups at 8 weeks compared to baseline. In the serum biochemical data, the total protein concentration was significantly (*p* < 0.05) higher in the AGE group than in the Placebo group at baseline and 4 weeks. The alanine aminotransferase activity was significantly (*p* < 0.01) lower in the AGE group than in the Placebo group at 8 weeks. Calcium concentration was significantly (*p* < 0.05) higher in the AGE group than in the Placebo group at baseline, and decreased significantly (*p* < 0.001) in the AGE group at 8 weeks compared to baseline. Sodium concentration was significantly (*p* < 0.05) higher in the AGE group than in the Placebo group at baseline and at 4 and 8 weeks.

### 3.3. Gingival index score

**Figure 1:**
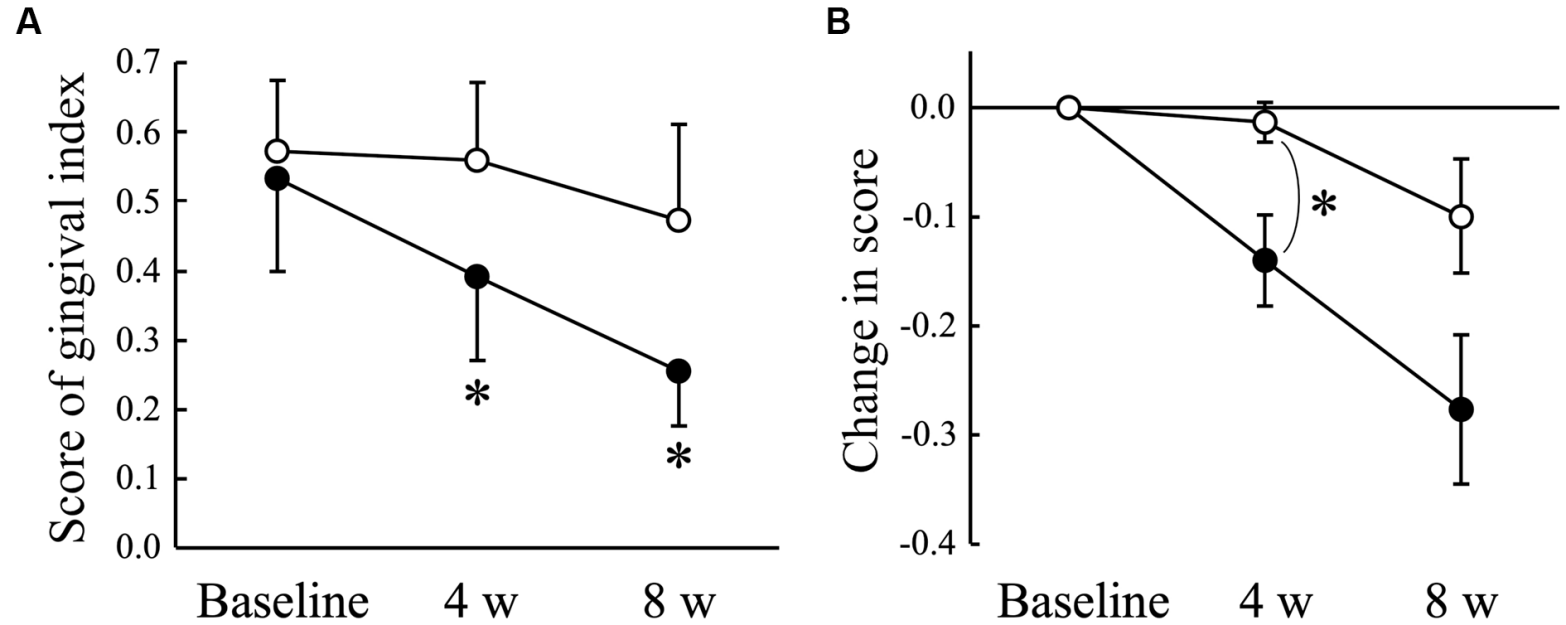
Gingival index score **(A)** and changed amount in the score **(B)** in Beagle dogs with mild gingivitis administered aged garlic extract (●, AGE group) and placebo power (○, Placebo group) for 8 weeks. Vertical bars indicate means ± standard error of the mean (*n* = 5). *p < 0.05, Wilcoxon signed rank test compared to baseline value, or Mann–Whitney U test compared between AGE and Placebo groups.

The average gingival index score obtained from 22 sites in the oral cavity decreased significantly (*p* < 0.05) in the AGE group at 4 (0.39 ± 0.12) and 8 weeks (0.25 ± 0.08) compared with that at baseline (0.53 ± 0.13), but not in the Placebo group during the experimental period (Figure 1A). There was a significant (*p* < 0.05) difference in the change from baseline between the Placebo and AGE groups at 4 weeks, but not (*p* = 0.095) at 8 weeks (Figure 1B).

### 3.4. VSCs level in exhaled air

**Figure 2:**
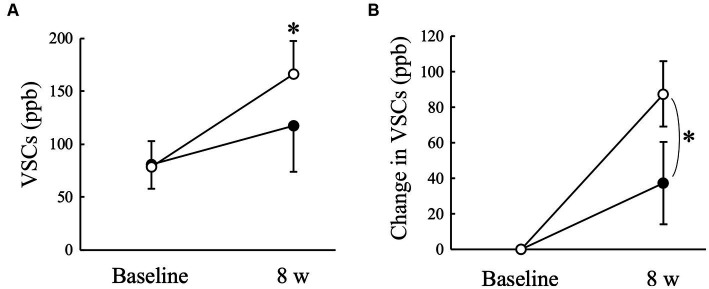
Level of volatile sulfur compounds (VSCs) **(A)** and changed amount in the level **(B)** in Beagle dogs with mild gingivitis administered aged garlic extract (●, AGE group) and placebo power (○, Placebo group) for 8 weeks. Vertical bars indicate means ± standard error of the mean (*n* = 5). *p < 0.05, Wilcoxon signed rank test compared to baseline value, or Mann–Whitney U test compared between AGE and Placebo groups.

**Figure 3:**
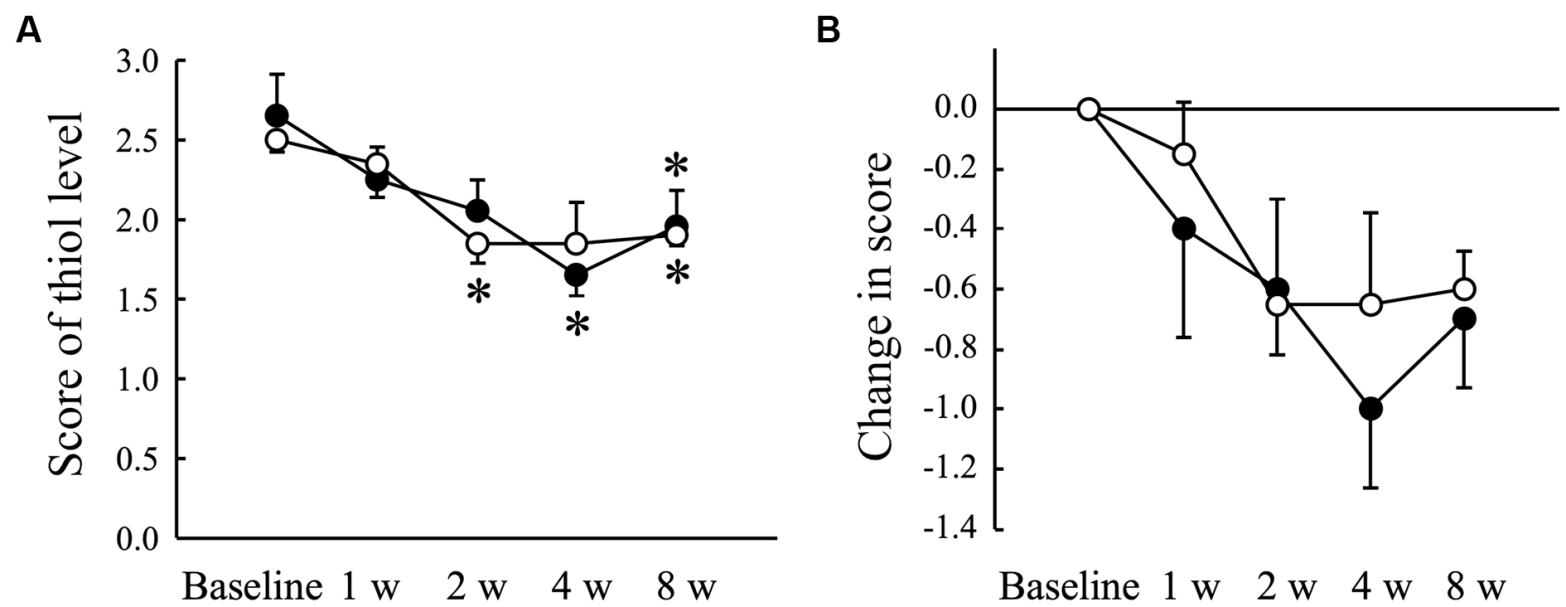
Score indicating the level of thiol, a volatile sulfur compound, measured using OraStrip test **(A)** and changed amount in the score **(B)** in Beagle dogs with mild gingivitis administered aged garlic extract (●, AGE group) and placebo power (○, Placebo group) for 8 weeks. Vertical bars indicate means ± standard error of the mean (*n* = 5). *p < 0.05, Wilcoxon signed rank test compared to baseline value.

The VSCs level of exhaled air measured using a halimeter increased significantly (*p* < 0.05) in the Placebo group at 8 weeks compared to that at baseline, but not in the AGE group (Figure 2A). There was a significant (*p* < 0.05) difference in the change from baseline between the Placebo and AGE groups at 8 weeks (Figure 2B). The average score indicating the thiol level estimated using OraStrip decreased significantly (*p* < 0.05) in the Placebo group at 2 and 8 weeks compared to baseline, and decreased significantly (*p* < 0.05) in the AGE group at 4 and 8 weeks compared to baseline (Figure 3A). However, there was no significant difference in the change from baseline between the two groups (Figure 3B).

### 3.5. Enzyme activity of periodontal pathogenic bacteria

**Figure 4:**
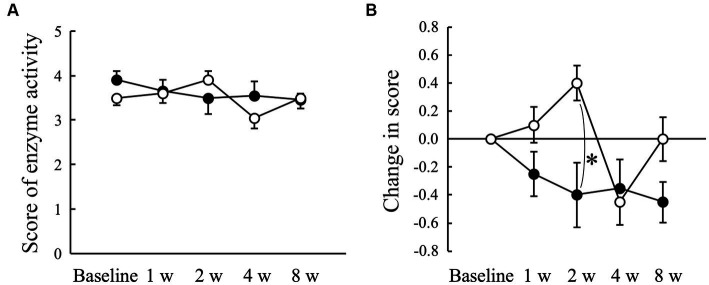
Score indicating the enzyme activity of periodontal pathogens measured using ADplit test **(A)** and changed amount in the score **(B)** in Beagle dogs with mild gingivitis administered aged garlic extract (●, AGE group) and placebo power (○, Placebo group) for 8 weeks. Vertical bars indicate means ± standard error of the mean (*n* = 5). *p < 0.05, Mann–Whitney U test compared between AGE and Placebo groups.

The average score obtained using ADplit showed no significant changes compared to baseline in both the Placebo and AGE groups (Figure 4A). However, the change from the baseline value tended to increase in the Placebo group at 2 weeks, whereas it tended to decrease in the AGE group during the experimental period (Figure 4B). There was a significant difference (*p* < 0.05) between the two groups at 2 weeks. The score was still lower in the AGE group than in the Placebo group at 8 weeks, but there was no significant difference (*p* = 0.095) between the two.

### 3.6. Salivary IgA and CAMP concentrations

**Figure 5:**
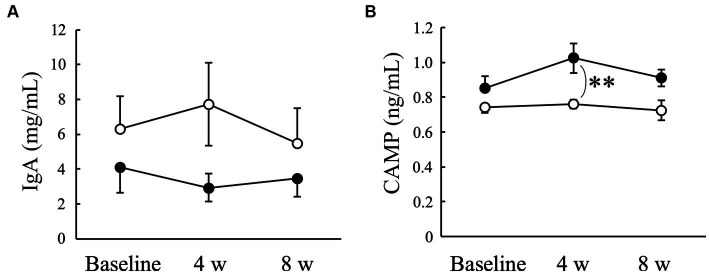
Concentrations of immunoglobulin A (IgA) **(A)** and cathelicidin antimicrobial peptide (CAMP) **(B)** in Beagle dogs with mild gingivitis administered aged garlic extract (●, AGE group) and placebo power (○, Placebo group) for 8 weeks. Vertical bars indicate means ± standard error of the mean (*n* = 5). **p < 0.01, Mann–Whitney U test compared between AGE and Placebo groups.

There was no significant change in the salivary IgA concentration in either the Placebo or AGE groups during the experimental period (Figure 5A). In contrast, the CAMP concentration tended to increase in the AGE group compared to the baseline value but not in the Placebo group (Figure 5B). There was a significant (*p* < 0.01) difference between the two groups at 4 weeks. The CAMP level was still higher in the AGE group than in the Placebo group at 8 weeks, but there was no significant difference (*p* = 0.056) between the two.

## 4. Discussion

Garlic intake induces the oxidation of erythrocytes and hemoglobin in dogs (47), resulting in hemolytic anemia (22). Therefore, we first examined the safety and adverse effects of AGE in Beagle dogs using the methods described in our previous study (39). Our previous study demonstrated no adverse effects, including hemolytic anemia, when AGE was orally administered to healthy dogs at 90 mg/kg/day for 12 weeks (39). Consistent with a previous study, the present study demonstrated that feeding of AGE at a low dose of 18 mg/kg/day for 8 weeks had no adverse effects on the general health of dogs with mild gingivitis (Supplementary Table S1). Although the administration of AGE induced statistically significant differences in several hematological and serum biochemical parameters, such as erythrocyte, leukocyte, and monocyte counts, and calcium concentration compared to baseline values, the differences were quite small, and all the parameters were kept within the reference values in Beagle dogs (48, 49). In our previous study, dogs administered AGE at 45 and 90 mg/kg/day for 12 weeks, the doses and duration of which were higher and longer than those in this study (18 mg/kg/day for 8 weeks), showed no significant changes in erythrocyte count and other hemolytic anemia-associated parameters including reticulocyte count, lactate dehydrogenase activity, total bilirubin concentration, and erythrocyte oxidation parameters such as Heinz body and eccentrocyte counts (39). Therefore, we considered the significant decrease in erythrocyte count at 4 and 8 weeks in the AGE group in this study to be incidental, innocuous, and unrelated to anemia. However, garlic has the potential for hemolytic anemia in dogs (22, 47), and therefore, further studies are necessary in order to confirm the complete safety of AGE for the use as a supplement.

The major objective of this study was to investigate the therapeutic effect of AGE on gingivitis in dogs using multiple evaluation indicators, including the gingival index score (Figure 1), VSCs levels in exhaled air (Figure 2), thiol levels (Figure 3), enzyme activity of periodontal pathogenic bacteria (Figure 4), and salivary IgA and CAMP concentrations (Figure 5). Based on the results obtained from these evaluation indicators, we considered that AGE has a potential therapeutic effect on canine gingivitis, as describe later.

The clinical signs of canine periodontal disease include gingival inflammation with redness, swelling, and bleeding (8). Therefore, the severity of gingivitis was evaluated using a gingival index score between 0 and 3, as previously employed in other studies (41, 42). The score significantly decreased in the AGE group only and was significantly lower in the AGE group than in the Placebo group at 4 weeks (Figure 1). The score in the AGE group continued to be lower than in the Placebo group at 8 weeks, although there was no significant difference. This suggests that AGE can improve the degree of gingival inflammation in dogs.

Oral malodor, also known as halitosis, is the first clinical sign of periodontal disease detected by dog owners (50). The major sources of halitosis are VSCs, including hydrogen sulfide, methyl mercaptan, and dimethyl sulfide, which are produced by anaerobic oral bacteria (51). Oral Gram-negative anaerobic bacteria can degrade proteinaceous components from saliva, blood cells, oral epithelial cells, and food debris into cysteine and methionine, resulting in the production of VSCs (52). VSCs levels in exhaled air correlate positively with clinical parameters of periodontal disease, such as gingival and calculus indices in Beagle dogs (5). In this study, the VSCs level increased significantly in the Placebo group and the OraStrip score decreased significantly in both groups regardless of the administration of AGE, possibly because the gingival inflammatory conditions of the examined dogs were still actively changing during the experimental period. In such a situation, the administration of AGE suppressed the increase in VSCs levels in the exhaled air (Figure 2). However, this study also showed that there was no significant difference in the OraStrip test score between the AGE and Placebo groups (Figure 3), which is associated with thiol levels at the gingival margin. A clinical study on human subjects indicated a relationship between halitosis and VSCs-producing microorganisms on the tongue (52). Oral bacteria associated with halitosis, such as *Porphyromonas*, *Fusobacterium*, and *Streptococcus* species, colonize the dorsal mucosa of the canine tongue (53). Taken together, these reports suggest that VSCs production in exhaled air results from not only the gingival marginal microflora but also the microflora on the tongue. Therefore, AGE-induced suppression of VSCs levels in canine exhaled air may be attributed to the prevention of halitosis-associated bacterial proliferation on the tongue. Further studies are needed to determine the effects of AGE on the tongue microflora.

Human periodontal pathogens such as *Porphyromonas gingivalis*, *Treponema denticola*, *Tanerella forthysia*, and *Capnocytophaga ochracea* produce trypsin-like enzymes that hydrolyze *N*-benzoyl-DL-arginine-2-naphthylamide (BANA) (54). Diagnostic aids that use BANA to indicate the presence of periodontopathic bacteria can be conveniently used at the chairside in human dental medicine and have been shown to correlate well with the clinical indicators used to diagnose periodontal disease (55, 56). Oral bacteria in dental plaque play an important role in the initiation and progression of gingivitis and periodontitis (3, 5). A cross-sectional survey reported that the prevalent pathogenic species identified in dogs with healthy gingiva, gingivitis, and mild periodontitis were *Peptostreptococcus*, *Peptostreptococcaceae*, and *Actinomyces* species, and that *Corynebacterium canis* was significantly more abundant in dogs with gingivitis and periodontitis than in healthy dogs (57). Another study revealed that the predominant pathogens were *Bacteroides heparinolyticus*, *Pasteurella dagmatis*, *Actinomyces canis*, *Porphyromonas cangingivalis*, and *Desulfomicrobium orale* in dogs with gingivitis or periodontitis (2). In particular, the hydrolytic activity of BANA by *Corynebacterium* and *Actinomyces* species is positively correlated with the severity of periodontal disease in Beagle dogs (58). In this study, the administration of AGE suppressed the hydrolytic activity of BANA compared with the placebo (Figure 4), although this is not direct evidence for periodontal pathogens. The *in vitro* studies demonstrated that ethanolic and aqueous garlic extracts inhibited the growth of human periodontal pathogens such as *Porphyromonas gingivalis* and *Aggregatibacter actinomycetemcomitans* (59). Diallyl sulfide, a lipophilic constituent in AGE, induces cell death in *Aggregatibacter actinomycetemcomitans* via glutathione *S*-transferase inhibition (60). These results suggested that garlic extract and garlic-derived phytochemicals possess direct antimicrobial activity against periodontal pathogens. However, the antimicrobial activity of AGE and its constituents, other than diallyl sulfide, against periodontal pathogens is unknown. Therefore, further studies, particularly in dogs, are required.

Saliva contains a wide variety of antimicrobial substances, and the maintenance of oral microflora by these substances plays an important role in preventing periodontal disease (61, 62). Antimicrobial substances in whole saliva are derived from various cells, such as epithelial cells, salivary glands, and neutrophils (61). Saliva is a rich source of oral epithelial cells that express functional toll-like receptors (TLRs) such as TLR-2 and TLR-4, which interact with most periodontal pathogens (63). IgA is commonly known as one of the first lines of defense against the adherence and invasion of pathogenic bacteria (64). Our findings indicated that AGE administration had no effect on salivary IgA concentrations (Figure 5A).

Antimicrobial peptides exhibit a broad spectrum of antibacterial activities (65) and play an important role in innate immune responses (66). Furthermore, antimicrobial peptides directly regulate the balance between pro- and anti-inflammation (65). These reports suggest that antimicrobial peptides contribute not only to the defense against oral pathogenic bacteria but also to the suppression of gingival inflammation, resulting in the prevention and treatment of periodontal disease. Cathelicidins are a family of antimicrobial peptides commonly found in numerous mammals such as humans, mice, and dogs (67). A previous proteomics study detected CAMP in the saliva of healthy dog (60). In this study, the administration of AGE increased the salivary CAMP concentration in dogs with mild gingivitis (Figure 5B), suggesting that the AGE-induced increase in salivary CAMP concentration might contribute partly to the suppression of gingivitis (Figure 1), VSCs levels in exhaled air (Figure 2), and enzyme activity of periodontal pathogens (Figure 4).

As mentioned, a clinical trial in humans demonstrated that the daily consumption of AGE for 4  months benefited oral health by reducing gingival inflammation and bleeding (36). An additional study in humans evaluated the loss of attachment caused by the formation of pockets between the tooth and gums in periodontitis following 18-month use of AGE, in which the level of periodontitis was significantly lower in the AGE group than in the placebo group (37). These two human clinical studies indicate that AGE may become significant in the prevention of periodontal diseases (68). However, the mechanisms through which AGE exerts its beneficial effects against periodontal diseases remain to be elucidated (68, 69). AGE may be a promising candidate for use in the treatment of periodontal diseases in humans, but further studies are required for the clarification of the basic molecular mechanisms involved (69). In this study, the duration (8 weeks) of use in dogs with gingivitis was shorter than those (4–18 months) in the human clinical studies, but there were significant outcomes including improved gingivitis and halitosis, suppressed hydrolytic activity of BANA, and increased salivary CAMP in dogs administered AGE. If the duration of administration is extended to a monthly or yearly level, the therapeutic effect of AGE on canine gingivitis may become clearer and the preventive effect of periodontal diseases may be exerted. An increased dosage (more than 18 mg/kg/day) may make the effect of AGE clearer. In addition, this study might suggest that the suppression of hydrolytic activity of BANA in periodontal pathogens and the increased concentration of salivary CAMP explain in part the therapeutic effects of AGE in periodontal diseases in animals and humans.

In this study, we examined the effects of AGE on gingivitis in Beagle dogs and discussed its usefulness. However, the major limitation was that the breed used was limited to Beagles and the sample size was small (five dogs in each group). A research group performed a large-scale study on the effects of *Ascophyllum nodosum* on canine oral health in 60 dogs of various breeds, including Japanese Chins, Miniature Schnauzers, Chihuahuas, Pomeranians, and West Highland White Terriers, discussing the evaluated dog breeds, body weights, and sample sizes (70). The assessment, in comparison with their previous results (71), revealed some differences among studies. Therefore, large-scale studies are needed to investigate the therapeutic effects of AGE on periodontal disease across a wide range of breeds and body sizes. Second, this study examined the effect of AGE only on mild gingivitis with gingival index of less than 1; thus, further studies are needed on dogs with more severe gingivitis and periodontitis.

In conclusion, the present study demonstrated for the first time that feeding of AGE at 18 mg/kg/day for 8 weeks improved gingivitis and halitosis in Beagle dogs with mild gingivitis. We also suggest that the direct antibacterial property of AGE and/or an increase in salivary CAMP may be involved in the underlying mechanism. These findings may support the potential application of AGE as an oral supplement for the prevention and treatment of gingivitis in dogs. Future studies will target the role of AGE in moderate to severe periodontal disease.

## Data availability statement

The original contributions presented in the study are included in the article/Supplementary material, further inquiries can be directed to the corresponding author.

## Ethics statement

The animal study was approved by the Animal Welfare Committee of Kitayama Labes Corporation (Approval Number NBC57-024; approval date: 28 January 2021). The study was conducted in accordance with the local legislation and institutional requirements.

## Author contributions

KT: Conceptualization, Data curation, Writing – original draft. HN: Data curation, Investigation, Writing – original draft. MU: Conceptualization, Data curation, Writing – original draft. MT: Investigation, Writing – review & editing. MN: Investigation, Writing – review & editing. TM: Investigation, Writing – review & editing. HJ: Conceptualization, Investigation, Methodology, Writing – review & editing. NA: Investigation, Writing – review & editing. SM: Investigation, Writing – review & editing. AY: Investigation, Writing – review & editing. YE: Supervision, Writing – review & editing. OY: Conceptualization, Investigation, Methodology, Resources, Supervision, Writing – review & editing.
