# Supplementary material for: Therapeutic effect of aged garlic extract on gingivitis in dogs
Source: Front Vet Sci. 2023 Nov 6;10:1277272. doi: 10.3389/fvets.2023.1277272 (PMC10658002; doi:10.3389/fvets.2023.1277272)
Supplement: Supplementary file 1 [file Table_1.docx]

Supplementary Material

# Supplementary Table 1.

# Changes in body weight, and hematological and serum biochemical parameters in Beagle dogs fed aged garlic extract (AGE; n=5) and not fed AGE (Placebo; n=5) for 8 weeks.

| **Parameter** | **Group** | **Baseline** | **Week 4** | **Week 8** |
| --- | --- | --- | --- | --- |
| Body weight | Placebo | 10.8 ± 0.41 | 10.5 ± 0.47 | 10.6 ± 0.48 |
| (kg) | AGE | 10.8 ± 0.35 | 10.4 ± 0.50 | 10.8 ± 0.54 |
| Statistics between groups | | NS | NS | NS |
| Erythrocyte | Placebo | 7.16 ± 0.21 | 6.68 ± 0.27 | 6.83 ± 0.31 |
| (X10^6^/µL) | AGE | 6.82 ± 0.27 | 6.17 ± 0.23* | 5.97 ± 0.35* |
| Statistics between groups | | NS | NS | NS |
| Hemoglobin | Placebo | 16.0 ± 0.38 | 15.1 ± 0.62 | 15.2 ± 0.64 |
| (g/dL) | AGE | 15.3 ± 0.43 | 14.2 ± 0.49 | 13.7 ± 0.67 |
| Statistics between groups | | NS | NS | NS |
| Hematocrit | Placebo | 48.4 ± 1.0 | 46.4 ± 1.8 | 46.2 ± 2.0 |
| (%) | AGE | 46.6 ± 1.4 | 43.8 ± 1.5 | 41.9 ± 2.2 |
| Statistics between groups | | NS | NS | NS |
| MCV | Placebo | 67.7 ± 0.92 | 69.6 ± 0.83 | 67.6 ± 0.87 |
| (fL) | AGE | 68.4 ± 0.99 | 70.9 ± 0.96 | 70.3 ± 1.13 |
| Statistics between groups | | NS | NS | NS |
| MCH | Placebo | 22.3 ± 0.31 | 22.7 ± 0.20 | 22.2 ± 0.32 |
| (pg) | AGE | 22.5 ± 0.29 | 23.1 ± 0.37 | 23.0 ± 0.49 |
| Statistics between groups | | NS | NS | NS |
| MCHC | Placebo | 33.0 ± 0.08 | 32.6 ± 0.22 | 32.9 ± 0.38 |
| (g/dL) | AGE | 32.9 ± 0.20 | 32.5 ± 0.17 | 32.8 ± 0.25 |
| Statistics between groups | | NS | NS | NS |
| Platelet | Placebo | 337 ± 41 | 313 ± 43 | 300 ± 34 |
| (X10^3^/µL) | AGE | 477 ± 67 | 430 ± 45 | 325 ± 25 |
| Statistics between groups | | NS | NS | NS |
| Leukocyte | Placebo | 8.86 ± 0.99 | 7.08 ± 0.30 | 6.92 ± 0.25* |
| (X10^3^/µL) | AGE | 8.40 ± 0.96 | 6.42 ± 0.70* | 6.60 ± 0.16 |
| Statistics between groups | | NS | NS | NS |
| Neutrophil | Placebo | 6.06 ± 0.11 | 4.64 ± 0.19 | 4.56 ± 0.21 |
| (X10^3^/µL) | AGE | 5.46 ± 0.67 | 4.02 ± 0.63 | 4.13 ± 0.19 |
| Statistics between groups | | NS | NS | NS |
| Lymphocyte | Placebo | 1.97 ± 0.22 | 1.74 ± 0.18 | 1.74 ± 0.14 |
| (X10^3^/µL) | AGE | 1.98 ± 0.25 | 1.65 ± 0.17 | 1.79 ± 0.16 |
| Statistics between groups | | NS | NS | NS |
| Monocyte | Placebo | 436 ± 61 | 371 ± 28 | 318 ± 35* |
| (/µL) | AGE | 520 ± 108 | 348 ± 43 | 308 ± 49* |
| Statistics between groups | | NS | NS | NS |
| Eosinophil | Placebo | 373 ± 62 | 298 ± 84 | 284 ± 55 |
| (/µL) | AGE | 411 ± 60 | 374 ± 49 | 348 ± 42 |
| Statistics between groups | | NS | NS | NS |
| Basophil | Placebo | 27.7 ± 3.3 | 33.6 ± 1.8 | 20.5 ± 1.6 |
| (/µL) | AGE | 29.0 ± 6.7 | 34.1 ± 5.2 | 26.5 ± 2.3 |
| Statistics between groups | | NS | NS | NS |
| Total protein | Placebo | 6.4 ± 0.15 | 6.3 ± 0.09 | 6.4 ± 0.15 |
| (g/dL) | AGE | 6.9 ± 0.11 | 6.7 ± 0.11 | 6.6 ± 0.20 |
| Statistics between groups | | *p* < 0.05 | *p* < 0.05 | NS |
| Albumin | Placebo | 3.7 ± 0.11 | 3.6 ± 0.10 | 3.6 ± 0.07 |
| (g/dL) | AGE | 3.8 ± 0.09 | 3.5 ± 0.12 | 3.5 ± 0.10 |
| Statistics between groups | | NS | NS | NS |
| Globulin | Placebo | 2.7 ± 0.12 | 2.8 ± 0.13 | 2.8 ± 0.17 |
| (g/dL) | AGE | 3.1 ± 0.14 | 3.2 ± 0.13 | 3.1 ± 0.17 |
| Statistics between groups | | NS | NS | NS |
| A/G ratio | Placebo | 1.35 ± 0.076 | 1.31 ± 0.084 | 1.31 ± 0.082 |
|  | AGE | 1.21 ± 0.073 | 1.13 ± 0.076 | 1.17 ± 0.078 |
| Statistics between groups | | NS | NS | NS |
| Glucose | Placebo | 94.4 ± 2.3 | 95.0 ± 1.7 | 92.6 ± 2.4 |
| (mg/dL) | AGE | 99.4 ± 2.2 | 93.0 ± 3.1 | 90.0 ± 2.9 |
| Statistics between groups | | NS | NS | NS |
| Amylase | Placebo | 430 ± 45 | 486 ± 63 | 446 ± 48 |
| (U/L) | AGE | 382 ± 34 | 466 ± 42 | 436 ± 38 |
| Statistics between groups | | NS | NS | NS |
| ALT | Placebo | 112.4 ± 61.4 | 65.6 ± 33.4 | 43.0 ± 2.5 |
| (U/L) | AGE | 65.6 ± 33.4 | 30.0 ± 2.0 | 30.6 ± 2.8 |
| Statistics between groups | | NS | NS | *p* < 0.01 |
| ALP | Placebo | 47.8 ± 9.0 | 51.4 ± 7.9 | 52.4 ± 7.5 |
| (U/L) | AGE | 46.2 ± 8.0 | 46.0 ± 2.6 | 42.2 ± 2.5 |
| Statistics between groups | | NS | NS | NS |
| Total bilirubin | Placebo | 0.30 ± 0.00 | 0.30 ± 0.00 | 0.38 ± 0.06 |
| (mg/dL) | AGE | 0.30 ± 0.00 | 0.30 ± 0.00 | 0.30 ± 0.00 |
| Statistics between groups | | NS | NS | NS |
| BUN | Placebo | 12.6 ± 0.87 | 11.6 ± 0.68 | 10.8 ± 0.20 |
| (mg/dL) | AGE | 14.0 ± 1.84 | 14.0 ± 1.70 | 12.6 ± 1.69 |
| Statistics between groups | | NS | NS | NS |
| Creatinine | Placebo | 0.78 ± 0.111 | 0.70 ± 0.114 | 0.74 ± 0.068 |
| (mg/dL) | AGE | 0.62 ± 0.058 | 0.54 ± 0.051 | 0.64 ± 0.081 |
| Statistics between groups | | NS | NS | NS |
| Total calcium | Placebo | 9.8 ± 0.12 | 9.7 ± 0.13 | 9.8 ± 0.13 |
| (mg/dL) | AGE | 10.3 ± 0.10 | 9.9 ± 0.10 | 10.0 ± 0.08^#^ |
| Statistics between groups | | *p* < 0.05 | NS | NS |
| Phosphorus | Placebo | 5.44 ± 0.19 | 5.10 ± 0.21 | 5.12 ± 0.20 |
| (mg/dL) | AGE | 5.38 ± 0.31 | 4.94 ± 0.12 | 5.12 ± 0.27 |
| Statistics between groups | | NS | NS | NS |
| Sodium | Placebo | 142 ± 0.51 | 141 ± 0.81 | 141 ± 0.60 |
| (mEq/L) | AGE | 145 ± 0.71 | 144 ± 0.73 | 145 ± 0.20 |
| Statistics between groups | | *p* < 0.05 | *p* < 0.05 | *p* < 0.01 |
| Potassium | Placebo | 4.6 ± 0.12 | 4.8 ± 0.12 | 4.7 ± 0.08 |
| (mEq/L) | AGE | 5.0 ± 0.24 | 4.9 ± 0.13 | 4.9 ± 0.17 |
| Statistics between groups | | NS | NS | NS |
| SAA | Placebo | 2.40 ± 1.13 | 1.26 ± 0.10 | 1.08 ± 0.05 |
| (mg/L) | AGE | 2.84 ± 0.78 | 1.97 ± 0.30 | 1.88 ± 0.33 |
| Statistics between groups | | NS | NS | NS |
| CRP | Placebo | 1.75 ± 1.26 | 0.98 ± 0.52 | 0.36 ± 0.15 |
| (mg/dL) | AGE | 1.48 ± 1.05 | 0.38 ± 0.16 | 0.22 ± 0.15 |
| Statistics between groups | | NS | NS | NS |

Data are presented as mean ± standard error from five dogs per group. MCV, mean corpuscular volume; MCH, mean corpuscular hemoglobin; MCHC, mean corpuscular hemoglobin concentration; A/G, albumin/globulin; ALT, alanine aminotransferase; ALP, alkaline phosphatase; BUN, blood urea nitrogen; SAA, serum amyloid A; CRP, C-reactive protein. **p* < 0.05 and #*p* < 0.001 compared with baseline data. NS: Not significant between the two groups.
